# Conservative approach for management of fractured maxillary central incisors in young adults

**DOI:** 10.1002/ccr3.3177

**Published:** 2020-08-23

**Authors:** Jose Villalobos Tinoco, Carlos Alberto Jurado, Mohammed E. Sayed, Jose Obed Garcia Cortes, Zinaida Kaleinikova, Alfredo Hernandez, Abdulrahman Alshabib, Akimasa Tsujimoto

**Affiliations:** ^1^ Department of Oral Rehabilitation Autonomous University of Queretaro School of Dentistry Queretaro Mexico; ^2^ Clinical Digital Dentistry A. T. Still University Arizona School of Dentistry and Oral Health Mesa Arizona USA; ^3^ Department of Prosthetic Dental Sciences Jazan University College of Dentistry Jazan Saudi Arabia; ^4^ Autonomous University of San Luis Potosi School of Dentistry San Luis Potosi Mexico; ^5^ Comprehensive Care Unit A. T. Still University Arizona School of Dentistry and Oral Health Mesa Arizona USA; ^6^ Prosthodontics Care A. T. Still University Arizona School of Dentistry and Oral Health Mesa Arizona USA; ^7^ Department of Restorative Dentistry King Saud University College of Dentistry Riyadh Saudi Arabia; ^8^ Department of Operative Dentistry Nihon University Tokyo Japan

**Keywords:** dentistry

## Abstract

Ceramic restorations could be an acceptable treatment choice for fractured central incisors. A successful esthetic and conservative result to restore damaged anterior teeth can be obtained through proper evaluation, diagnostic wax‐up, guided minimal preparations, ceramic selection, and bonding protocols. Handcrafted glass‐based restorations can mimic contours and shape of natural teeth.

## Graphical abstract

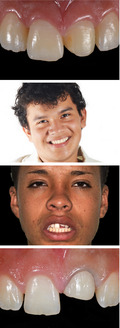

## 1 INTRODUCTION

Dental trauma is a common reason for tissue loss. Rehabilitation options for fractured incisors depends on the injuries' characteristics. Restoring teeth in the esthetic area may represent a challenge; however, adhesive materials and ceramic restorations may present a healthful solution, because they offer minimally invasive properties and excellent esthetic appearances.

Dental trauma to the frontal teeth is a common problem among teenagers and young adults.^1^ The most common reasons of these type of injures are accidents and sport activities.^2^ The hardness and direction of the impact are factors determining the surrounding tissue and tooth damage.^3^ Every time the anterior region is involved, it is important to satisfy esthetic requirements, since a delightful smile looks to play a key psychosocial role in young adults' life and relationships.^4^ Therefore, a thorough clinical examination is crucial for determining the appropriate treatment option.^5^ During a clinical evaluation, extraoral and intraoral situations need to be carefully observed. In addition, patient's radiographic examination, medical, and dental history, vitality test, dislocation of teeth, damage of the periodontal tissues and percussion analysis findings must be considered.^6^ In some situations the treatment needs to be provided quickly otherwise the prognosis could be compromised over the time.^7^ In case, a complete dental fragment is available and it is in good conditions, reattachment may be a possibility.^8^, ^9^

When selecting the type of treatment, some clinical aspects are key to be considered, like the quality of the remaining tooth structure, location of the fracture line, age of the patient, and the presence of parafunctional habits.^10^, ^11^ Despite the fact that composite resins are an option for these clinical situations, all‐ceramic restorations as a treatment possibility can provide high esthetic results that show resistance to the loss of shine, shade, and longevity,^12^ which is a limitation of the direct restorative materials.^13^ The opportunity of etching the ceramic surface significantly improved the long‐term effectiveness of bonding to composite materials and tooth tissues.^14^, ^15^, ^16^, ^17^ All‐ceramic restorations can preserve dental integrity by using partial preparations such as veneers that reduces only a half to a quarter of the tooth compared with traditional crowns.^18^

The preparation design required for glass‐based ceramic restorations is different from traditional preparations, such as porcelain fused to metal or full gold, because nonadhesive restorations require designs to facilitate mechanical retention. Bonded ceramics do not require extensive tooth preparation, therefore sound dental structure can be preserved.^18^, ^19^, ^20^ Ceramic restorations are often mentioned to be the material of choice due to their higher fracture resistance and color stability.^12^

Glass‐ceramic material has physical characteristics similar to tooth enamel,^21^ and it has been demonstrated that if the lost enamel layer is reconstructed with porcelain veneers, the tooth totally regains its structural flexibility reaching values that are compared to an integral tooth.^22^ Tissue response to the presence of glass‐ceramics veneer is optimal^23^ with no reports known of toxic effects of breakdown products of dental porcelain.^24^ Therefore, the aim of this article is to report the conservative and esthetic clinical approach to fractured maxillary central incisors in two young adults.

## 2 CLINICAL REPORTS

### 2.1 Case one

**Figure 1:**
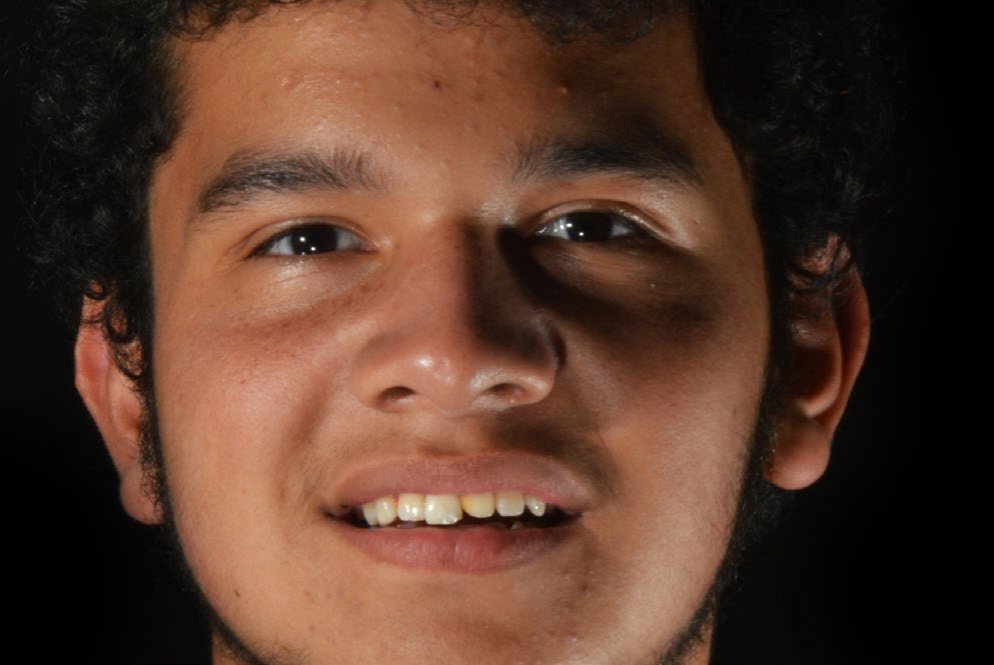
Initial face smiling

**Figure 2:**
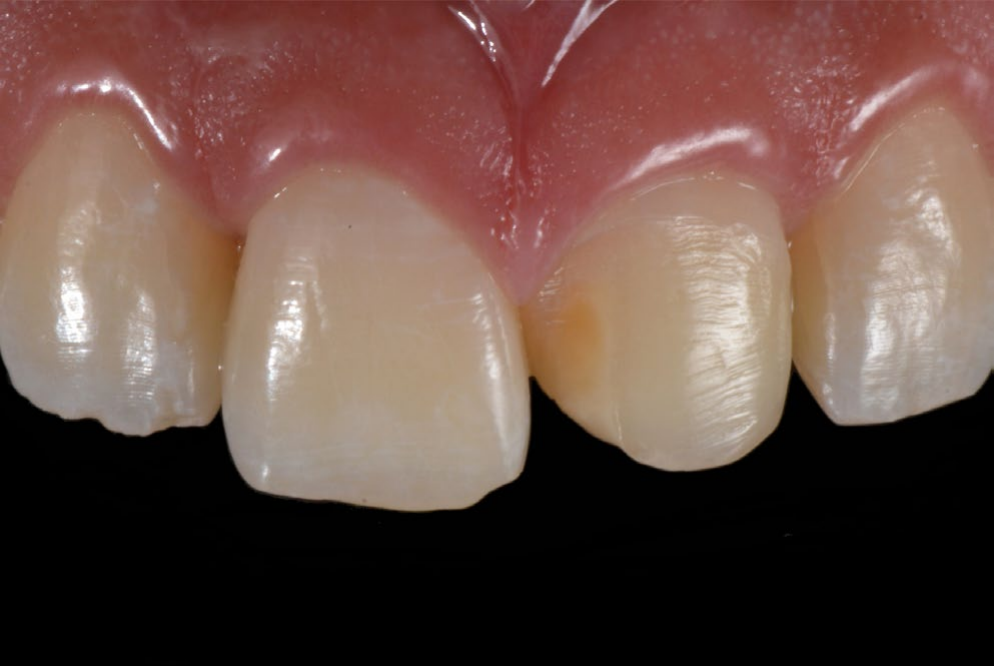
Initial intra‐oral

**Figure 3:**
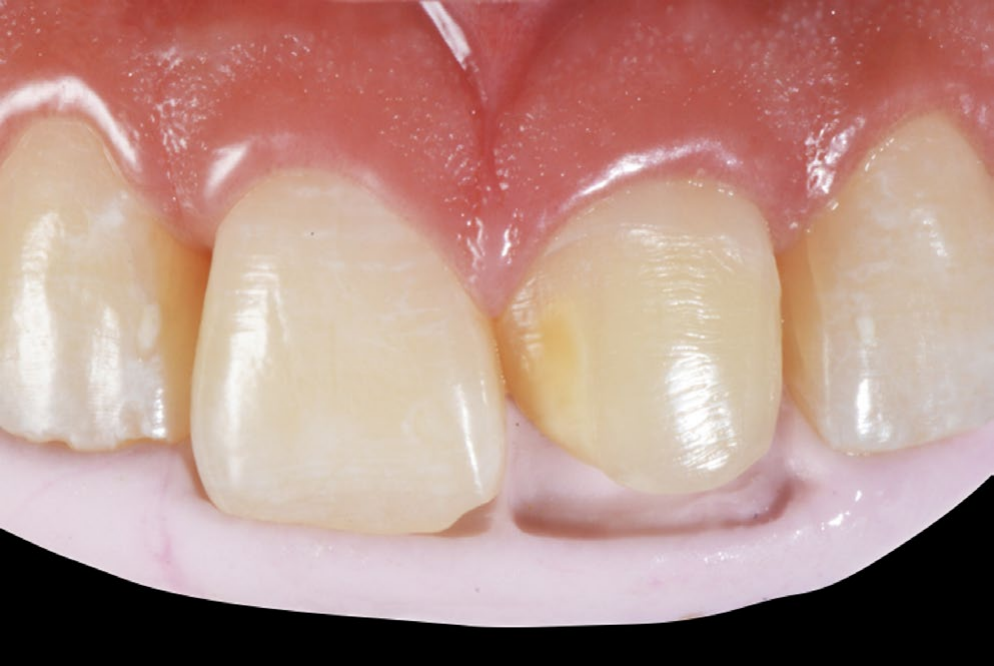
Matrix placed intra‐orally

**Figure 4:**
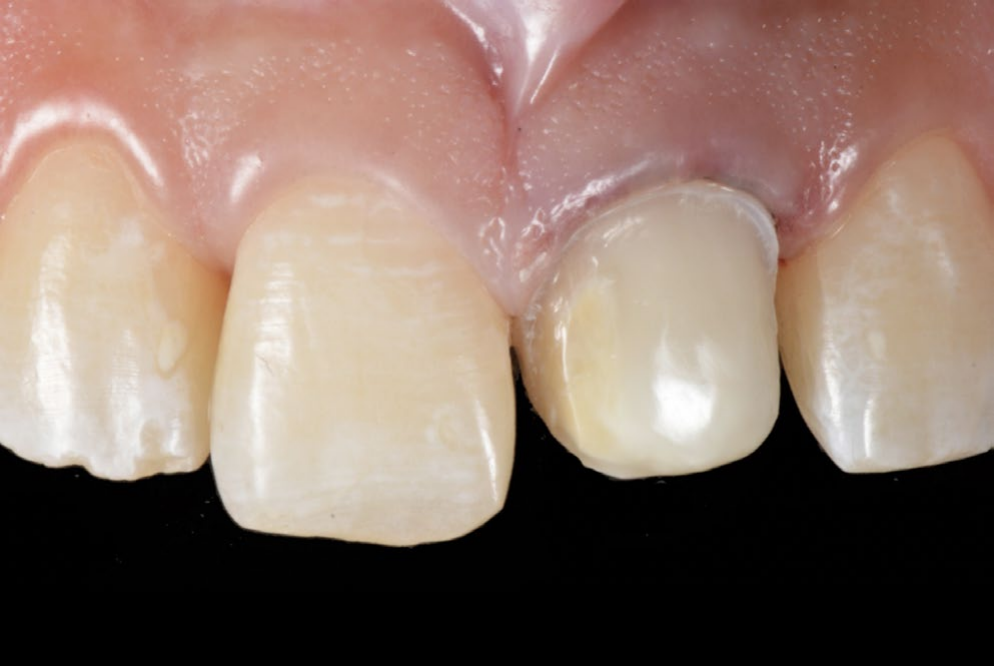
Minimal tooth preparation

A 15‐year‐old patient presented with his mother with the chief complaint “I would like to know how you can fix this broken tooth”. Patient had a bicycle accident, and then, he visited his nearest dentist for evaluation, patient was offered to have a full porcelain fused to metal crown to restore the broken tooth #9, however his mother disagreed with that option and stopped the treatment, she decided to look for more options. The mother has had history of full porcelain fused to metal crowns in the anterior segment and she dislikes the esthetic outcome and recalls that her teeth needed to have an aggressive preparation so she desires a more conservative and esthetic approach for her son. After a detailed radiographic and clinical assessment, the diagnosis was fracture of the facial, incisal, and mesial segment of tooth #9 and fracture of the incisal‐mesial line angle of tooth #8. (Figures 1 and 2) Patient and mother were offered a feldspathic veneer restoration for #9 and direct composite for #8. The mother was explained that the restorations will be highly esthetic due to the properties of feldspathic material and it will present a conservative approach with minimal tooth preparation based on the diagnostic wax‐up reduction guides. The patient and his mother approved the given treatment plan and requested to move to the following step of treatment. Diagnostic impressions were made with polyvinyl siloxane (Virtual, Ivoclar Vivadent) poured with type IV stone (Fujirock, GC America), followed by facebow record and diagnostic mounting on an articulator (Artex CR Amann, Girrbach). The patient was informed regarding the need to have a diagnostic wax‐up (GEO Classic Renfert) followed by fabrication of mock‐up guide in order to transfer the information to the mouth with a self‐cured temporary composite material (Bisacril Telio CS C&B Ivoclar Vivadent). When the diagnostic mock‐up was placed in mouth the smile line, occlusion, phonetics, and esthetics were evaluated. The patient was pleased with the visual and tactile result of temporary composite material, patient, and mother requested to initiate the treatment. A putty guide fabricated based on the diagnostic wax‐up was placed intraorally in order to evaluate the space available for the ceramic restoration (Figure 3) and minimally invasive tooth preparation was carried out only to smoothen the incisal edge and place a gingival margin for the future veneer on tooth #9. The final preparation was polished with polishing disks (Sof‐lex TX Disc, 3M) (Figure 4). A single cord impression technique with size #00 (Ultrapak, Ultradent Products Inc) and the final impression was made using light body and heavy body polyvinylsiloxane (Elite HD, Zhermack, Italy). The master cast and dies were fabricated with type IV stone (Fujirock, GC America Inc). Ceramic veneer was fabricated out of feldspathic porcelain (IPS e‐max, Ivoclar Vivadent). Tooth #8 surface was treated with 37% phosphoric acid etching gel for 15 seconds and gently dried for 5 seconds, followed by the adhesive agent application (Tetric N‐Bond Universal, Ivoclar Vivadent) for 20 seconds, removal of excess adhesive agent was done by gentle air and light curing was performed for 20 seconds (Valo LED, Ultradent). Composite (IPS Empress Direct, Ivoclar Vivadent) translucent and A1 shades were placed and light cured (Valo LED, Ultradent) for 20 seconds on each of the facial, mesial, incisal, palatal surfaces, independently. The first polishing of the surface was carried out using green and gray composite polishers (Composite Diamond Polisher, Jota), with application of polishing paste (Diamond Polish Mint, Ultradent) using a polishing brush (Jiffy Composite Polishing Brush, Ultradent), while the final polishing treatment was performed on the facial surface line angles with polishing disks (Sof‐Lex XT Disc, 3M) and polishing wheels (Polishing Composite, Kit 1921, Jota).

**Figure 5:**
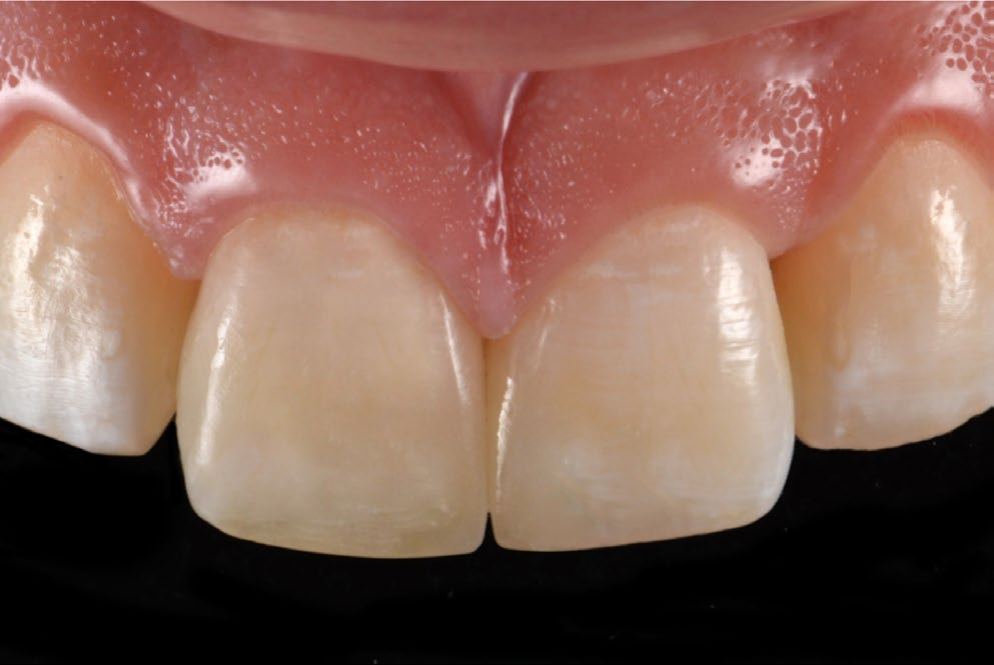
Final restorations

**Figure 6:**
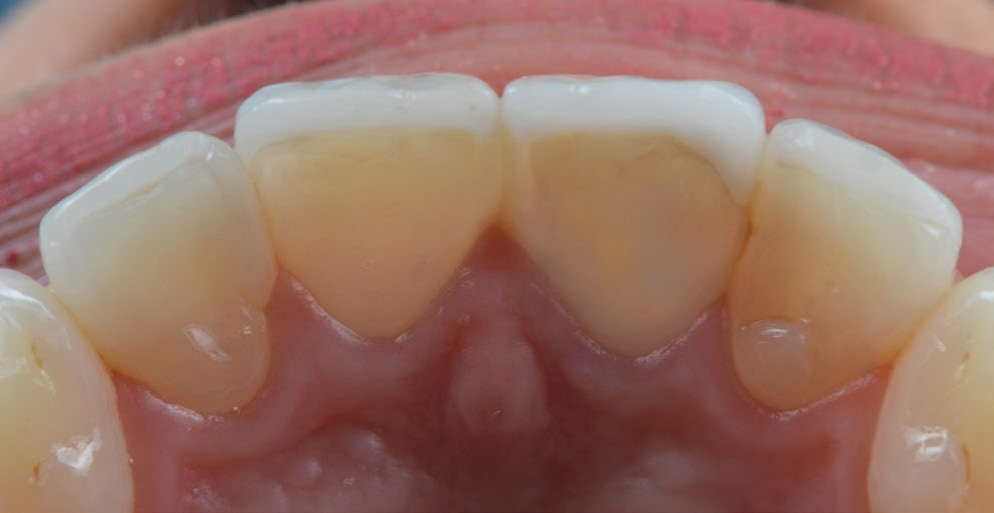
Final lingual view

**Figure 7:**
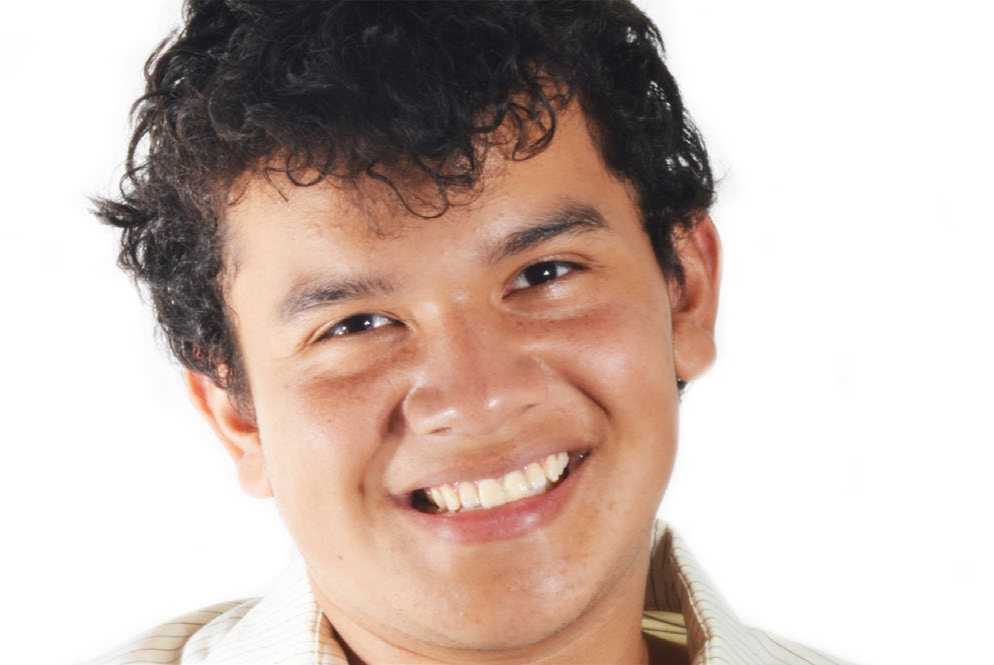
Final face smiling

**Figure 8:**
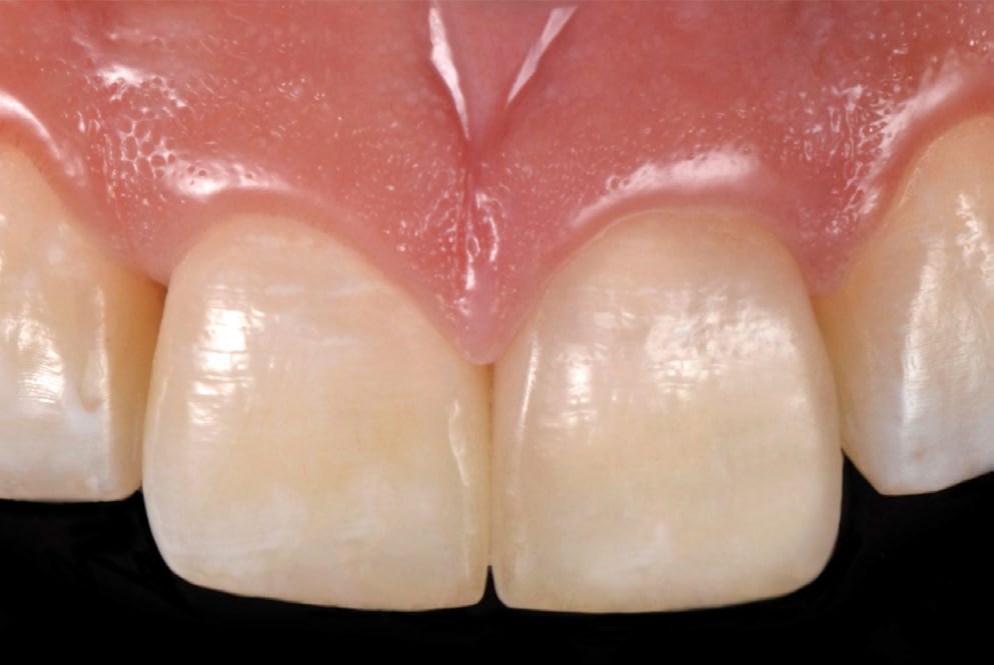
Two‐year follow‐up

The try‐in of ceramic restoration was performed, contours and margins were evaluated, and the patient and mother requested to proceed with the final bonding procedures. The tooth was cleaned with a pumice paste and chlorhexidine gluconate (Consepsis Scrub, Ultradent Products) in order to clean debris while disinfecting the area prior bonding. The ceramic restoration received hydrofluoric acid surface treatment (Porcelain Etch, Ultradent Products Inc) for 60 seconds, followed by rinsing and air‐drying. Restoration was submerged in water and alcohol in an ultrasonic bath (5300 Sweep Ultrasonic Cleaner, Quala Dental Products) for 5 minutes in order to remove any remaining acid. Next, silane (Monobond‐S, Ivoclar Vivadent) applied for 60 seconds followed by air‐drying. Cord of size #00 was packed around tooth #9 and the prepared surface was first treated with 37% phosphoric acid gel (Etch‐37 w/BAC, Bisco Dental) for 15 seconds and then rinsed and gently dried. The primer was applied (OptiBond FL, Kerr Dental) with excess being removed by air, followed by light curing (VALO LED Curing light, Ultradent Products Inc) for 20 seconds. The light‐cured luting cement (Variolink Veneer Neutral Shade, Ivoclar Vivadent) was applied to the ceramic veneer #9, which was placed against the prepared tooth surface with gentle vibrating finger pressure. The excess cement was removed with a micro‐brush and floss in the interproximal surfaces before light curing for 3 seconds on each of the facial, mesial, distal, and incisal surfaces, independently. Excess of cement in the cervical area was removed with a #12 scalpel blade (Surgical Scalpel Blade no. 12, Salvin Dental Specialties). Glycerin gel was then applied to the ceramic surface in order to prevent an oxygen inhibition layer (Deox, Ultradent Products Inc), and the surfaces were again light cured for 20 seconds each. Occlusion, protrusive, and excursive movements were evaluated with articulating paper (Articulating Paper Strips, Henry Schein). The patient was pleased with the final result (Figures 5, 6, 7). An acrylic resin occlusal device was provided to use at night in order to protect the patient's teeth and restorations. At the two‐year follow‐up, the patient was still pleased with the clinical outcome (Figure 8).

### 2.2 Case two

**Figure 9:**
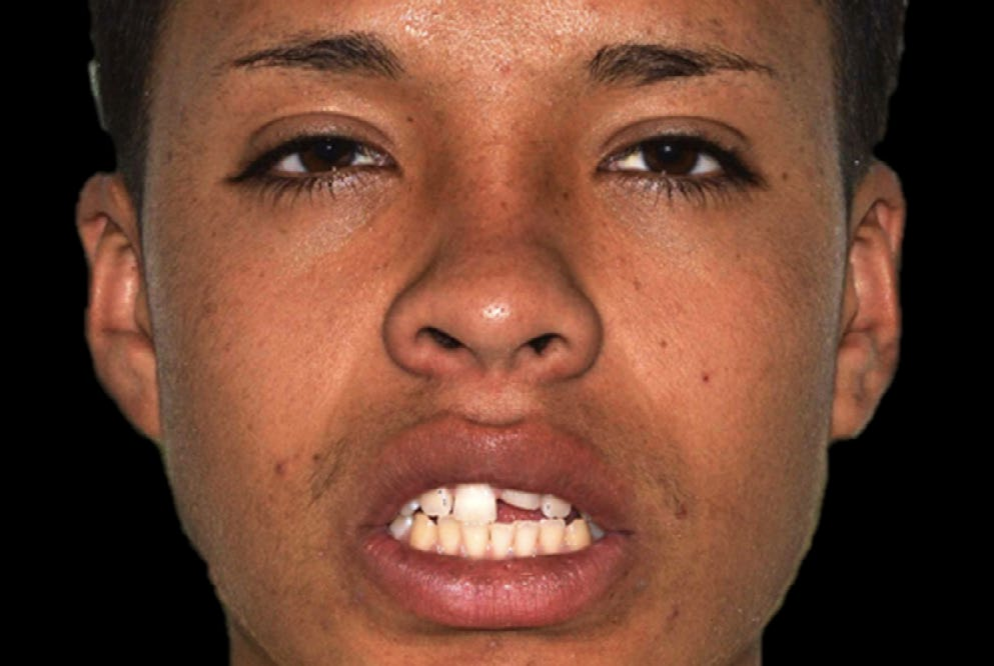
Initial face smiling

**Figure 10:**
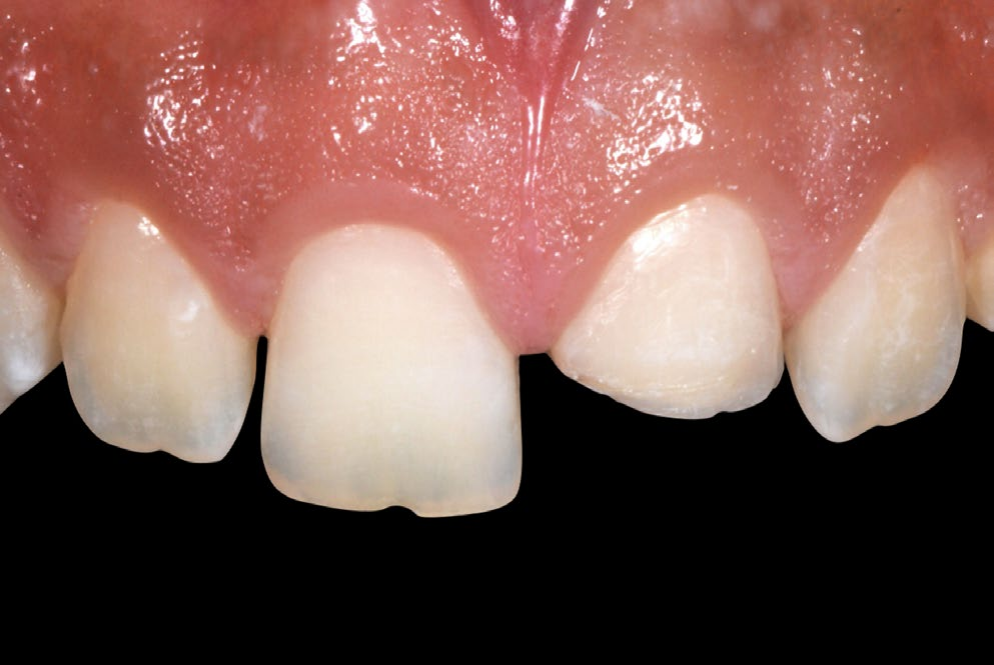
Initial intra‐oral

**Figure 11:**
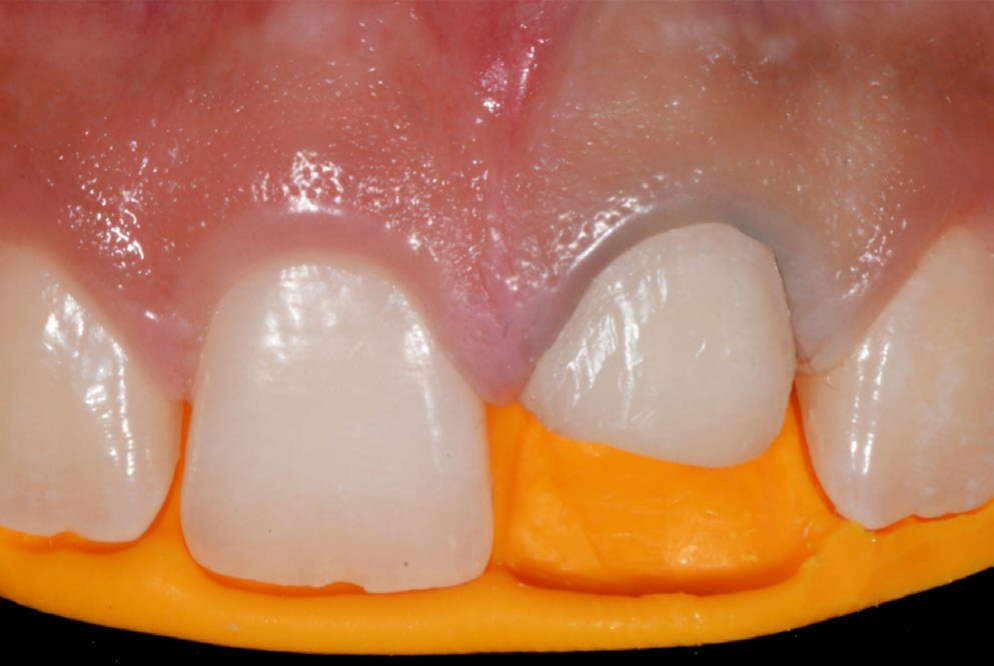
Minimal tooth preparation and reduction guide

**Figure 12:**
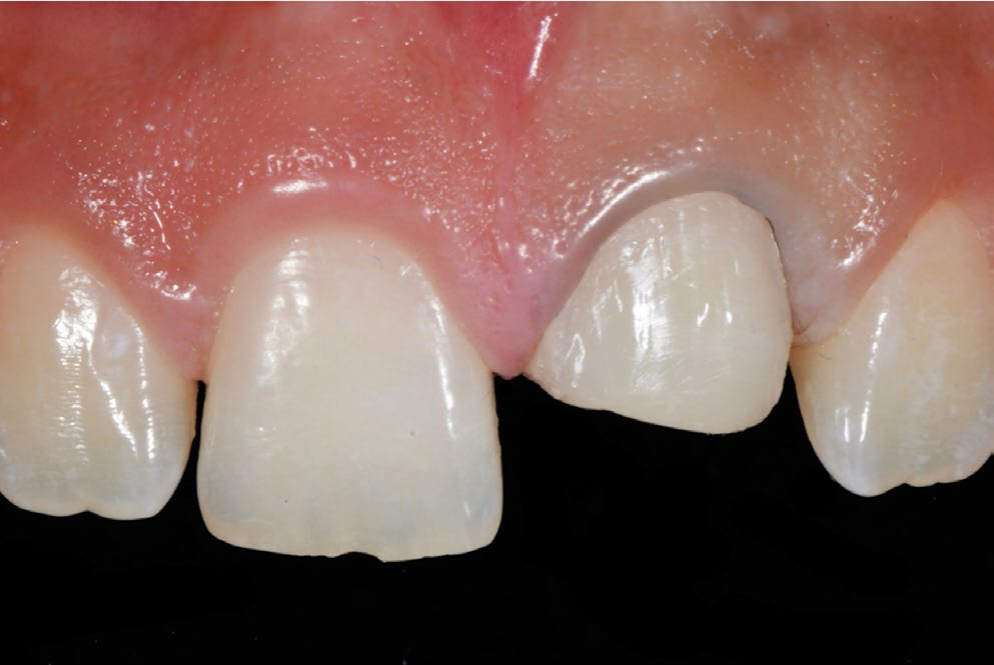
Final tooth preparation

A 13 years of age patient presented with his parent with the chief complaint of “I need to fix my broken tooth” (Figure 9). Patient had a trauma while playing baseball with his friends. After radiographic evaluation and clinical assessment, the diagnosis was fracture of tooth #9 (Figure 10). Patient was presented a minimally invasive treatment that included conservative tooth preparation for feldespathic veneer on #9, and patient and mother were informed that a very conservative preparation will only include a margin to seat the restoration without shortening of the tooth length. Traditional techniques including a facebow record and polyvinyl siloxane impressions (Virtual, Ivoclar Vivadent) were used for a diagnostic mounting (Artex CR Amann, Girrbach). Patient was explained that the diagnostic wax‐up and mock‐up would provide information about the tentative outcome. Diagnostic wax‐up (Wax GEO Classic, Renfert) was sculpted in order to provide a harmonious smile, taking into consideration the patient's desires and preferences. After presentation of the diagnostic wax‐up to the patient, a diagnostic mock‐up was performed with temporary bis‐acrylic material (Structur Premium, VOCO). Smile line, occlusion, phonetics, and esthetics were evaluated once the mock was placed. The patient and his mother liked the initial result and consented the treatment. Following diagnostic mock‐up removal, conservative tooth preparation was performed using different types of reduction guides (Figure 11). The final tooth preparation was polished using polishing disks (Soft‐lex TX Disc, 3M) (Figure 12). Final impression was made using a single cord of size #00 (Retraction Cord Plain Knitted, Ultrapak), impression trays (Rim‐Lock Impression Trays, Dentsply Caulk) were loaded with PVS in heavy body and light body consistency (Virtual 380, Ivoclar Vivadent) and final impression was made.

**Figure 13:**
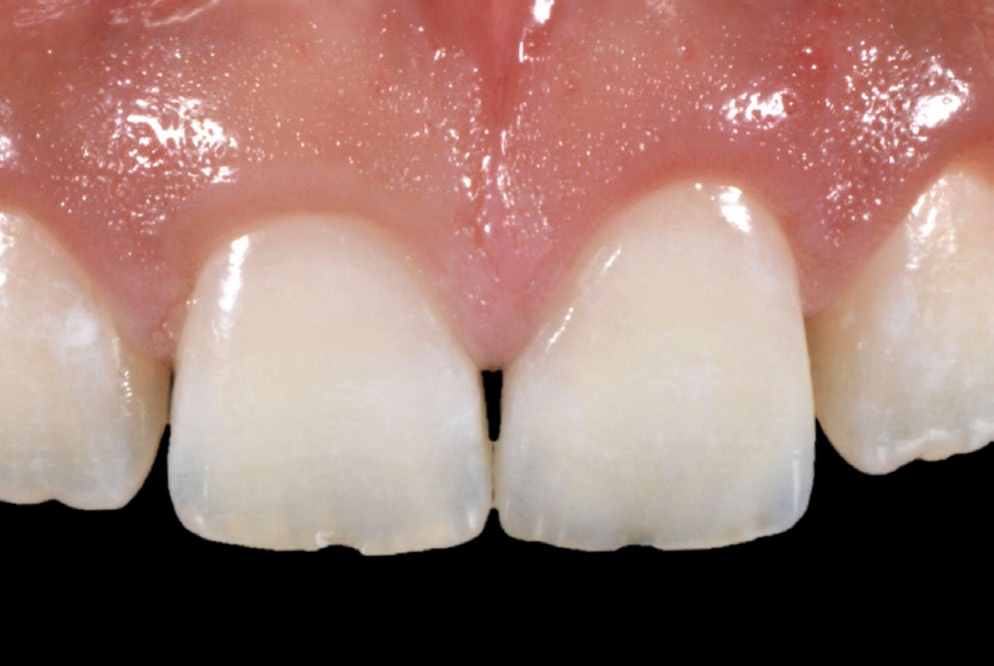
Final restoration

**Figure 14:**
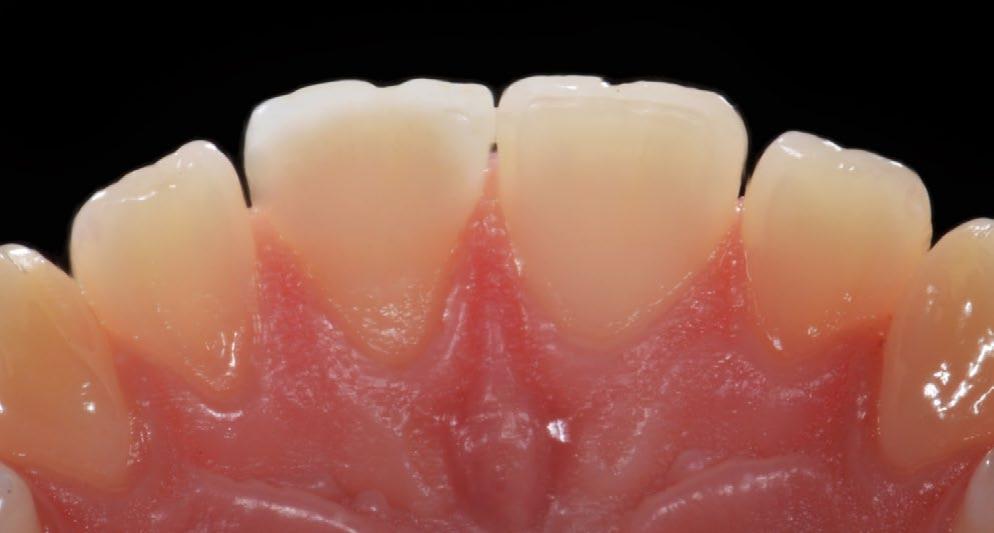
Final lingual view

**Figure 15:**
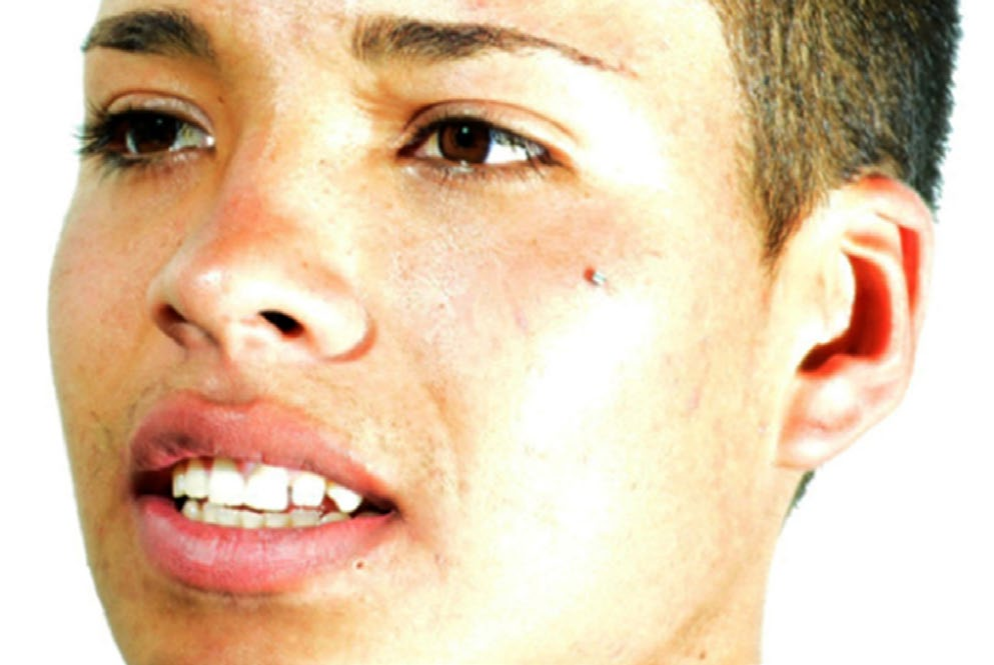
Final face smiling

**Figure 16:**
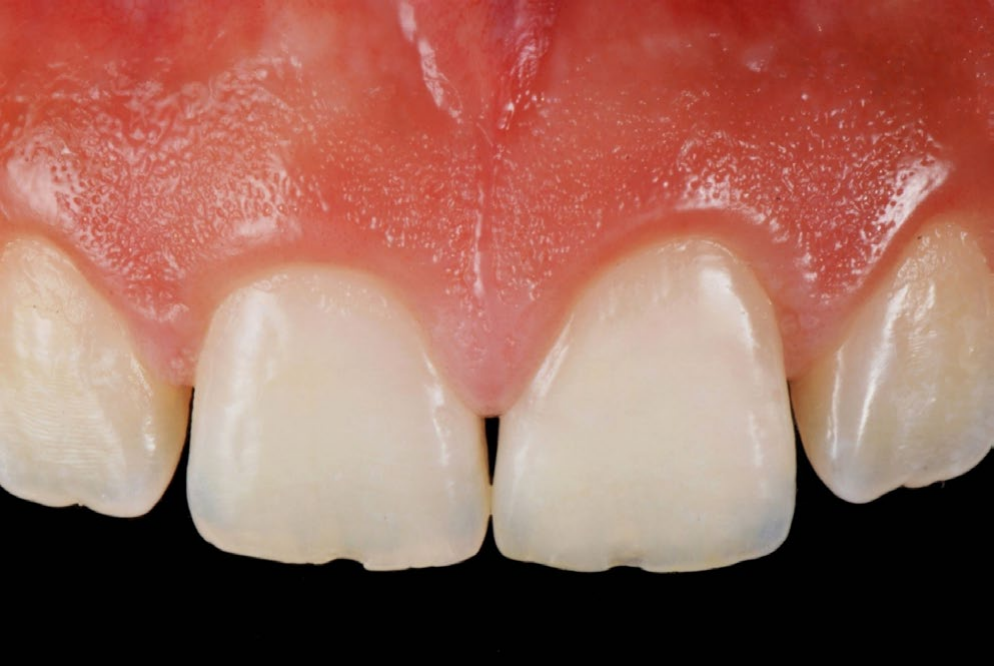
Two‐year follow‐up

The master cast was fabricated in type IV stone (Fujirock GC America) and feldespathic porcelain veneer was fabricated (Noritake Super Porcelain EX‐3, Kuraray Dental). A dry try‐in of the restorations was performed in order to evaluate the fit and contours of the final restoration, and once the patient and his mother approved the result, the bonding procedure continued. The ceramic restoration received hydrofluoric acid surface treatment (Porcelain Etch, Ultradent Products Inc) for 60 seconds, followed by rinsing and drying. Restorations were submerged in water and alcohol in an ultrasonic bath (5300 Sweep Ultrasonic Cleaner, Quala Dental Products) for 5 minutes in order to remove any remaining acid. Then silane (Monobond‐S, Ivoclar Vivadent) was placed for 60 seconds followed by air‐dried. A cord of size #00 was packed, and the tooth surface was first treated with 37% phosphoric acid gel (Etch‐37 w/BAC, Bisco Dental) for 15 seconds and then rinsed and gently dried. The primer was applied (OptiBond FL, Kerr Dental) and excess was removed by air, followed by light curing (VALO LED Curing light, Ultradent Products Inc) for 20 seconds. The light‐cured luting cement (Variolink Veneer Neutral Shade, Ivoclar Vivadent) was applied to the ceramic veneer, which was placed onto the tooth, and the excess cement was removed with a micro‐brush and floss in the interproximal surfaces before light curing for 3 seconds on each of the facial, mesial, distal, and incisal surfaces, independently. Excess cement removal, final curing, and occlusal adjustment were carried out following the same steps used for the first case. The patient and his mother were pleased with the end results (Figures 13, 14, 15), and an occlusal device was provided to use at night in order to protect the patient's teeth and restorations. At the two‐year follow‐up, the patient and his mother were also pleased with the treatment outcome (Figure 16).

## 3 DISCUSSION

Excessive dental tissue loss can occur because of trauma which causes esthetic, functional, and physiological problems.^25^ While elimination of the pain is priority, esthetic concerns are now gaining prominence.^26^ A number of treatment options have been proposed for crown fractures, each with their own advantages and disadvantages.^27^, ^28^, ^29^, ^30^, ^31^ Extraction of the fractured teeth should not be the first choice of treatment in young permanent tooth in the anterior area, ^32^ because it leads to bone loss in the area and compromise future implant therapy as a treatment,^33^ but if the remaining amount of tooth structure is not able to receive a fixed restoration and patient could not be qualified for orthodontic extrusion, then implant therapy could benefit the patient. Fortunately, the amount of tooth fractured in both patients was not large enough to consider the extraction option.

For uncomplicated tooth fractures, other approaches such as direct and indirect composite restorations have been suggested.^34^, ^35^ If a large amount of tooth structure needs to be replaced, then the indirect restoration may be suggested because it results in better proximal and occlusal contacts, better wear and marginal leakage resistance and enhancement of mechanical properties compared to direct composite restorations.^36^ However, ceramic veneers are also well known to be as a conservative treatment option for anterior teeth presenting fractures, wear, interdental spaces, and facial defects.^37^, ^38^ Bonded ceramic veneers have been proven to show reliable outcomes with positive long‐term results.^39^, ^40^, ^41^ The success of ceramic veneer restorations is based on many factors such as preparation design,^39^ adhesive techniques,^42^, ^43^ and adequate patient home care.^44^ With the new laboratory techniques and optimal dental materials, it is possible to produce ultrathin feldspathic veneers with a thickness of 0.1‐0.5 mm which can be bonded to a tooth structure with minimal or no preparation^45^, ^46^ in order to restore the fractured teeth. Since restorations do not last forever, conservative tooth preparation such as ceramic veneers prevent excessive removal of tooth structure and a second opportunity to the tooth for the future in case a secondary restoration is needed such as full coverage crown. Restorations provided for both cases were handcrafted because of the esthetic difficulty of matching the adjacent tooth and fabricating ultrathin restorations, especially for the first case, could represent a challenge with the current CAD/CAM technology.

Ceramic veneer preparations can be challenging for novice clinicians with limited experience, and the lack of good clinical protocols may result in failed restorations. The fabrication of proper diagnostic wax‐up is key for the diagnosis and treatment of fractured teeth with veneer restorations.^47^ It can provide information related to the discrepancies between the healthy and fractured tooth size, restorative space available, occlusal scheme, and any other treatment needed in the opposing arch.^48^, ^49^, ^50^ The diagnostic wax‐up can be transferred to the mouth as a diagnostic mock‐up and the patient can have a tactile and visual evaluation of the proposed restorations. The diagnostic wax‐up can also be used as treatment tool because it can be used to fabricate diagnostic and preparation guides. Diagnostic guides will allow the clinician to determine the thickness of the future restoration that replaces the fractured tooth segment and the reduction guide may help the clinician to reduce the extension of tooth preparation in case it is needed. The reduction guides were used for the planning and execution of final restorations in both cases. The diagnostic wax‐up helped us to provide a diagnostic mock‐up, in which patients and clinicians were able to visualize and have a tactile and visual evaluation of the proposed restorations, and then guides fabricated from the diagnostic wax‐up helped to evaluate the facial space for the future final restorations.

The formulation of a dental biomaterial with properties similar to actual dental tissue is a major aim of dental sciences. However, there are many obstacles faced in this task since the dental tissues found in humans are extremely complex and behave in specific and complicated ways during mastication. The material that currently most closely resembles human enamel is ceramic, which has many similar physical properties. Likewise, composite resin is the material that most closely resembles dentin.^51^, ^52^ It may seem logical to combine composite and ceramic materials, but there are a number of challenging factors to consider when formulating a novel biomaterial. An effective biomaterial needs to not only exhibit the esthetic and mechanical properties of a human tooth, but also must easily be manipulated and positioned.

The development of polymers reinforced with ceramic was an attempt to combine the durable and esthetic nature of ceramic with the versatile characteristics of composite resins, utilizing the amalgamation of different ceramics and polymers to achieve this goal. Innovative processing methods, including CAD/CAM technology, were used to design completely new ceramic‐reinforced polymers. CAD/CAM milling is used on industrial polymerized premade blocks to create these polymers. The blocks have been shown to have more structural reliability than restorations made by hand; the reason for this is that the high heat and pressure involved in the industrial polymerization process enhances monomer conversion and improves the cross‐linked matrix, which results in more desirable mechanical behavior.

There is widespread use of glass‐based ceramics in the development of dental restorations as there have been significant improvements in processing methods and, therefore, the mechanical properties of the material themselves have also improved. These enhanced mechanical properties are reflected in the durability of these restorations.^53^ The material's exceptional esthetics are another reason for glass‐ceramics' widespread use in restorations.

Materials with various compositions have been formed since glass‐ceramics were introduced to dentistry, but these materials became more popular after the introduction of lithium disilicate glass‐ceramic in 1998 (IPS Empress® 2, Ivoclar Vivadent Ltda, Schaan, Liechtenstein, later on marketed as e.max®). In comparison with feldespathic, Ceramic‐reinforced polymers and leucite glass‐ceramics, lithium disilicate‐based materials have shown to have superior mechanical properties (Table 1).^54^, ^55^, ^56^

**Table 1:** Mechanical properties for the most common glass‐ceramics in the market.^54^, ^55^, ^56^

Material	Flexural strength (MPa)	Fracture toughness (MPa)
Vita Enamic	150	1.72
Feldspathic glass‐ceramic	90	1.19
Leucite glass‐ceramic	160	1.03
Lithium disilicate glass‐ceramic	360	2.80

## 4 CONCLUSION

With the improvements in laboratory techniques, ultrathin handcrafted ceramic restorations can provide a highly esthetic and very conservative treatment option to rehabilitate a fractured central incisor. Adhesive ceramic restorations have been proven to show reliable outcomes with positive long‐term results. Based on the two case reports of fractured central incisors, the restorations were found to be clinically successful and fulfilled patients esthetic demands.

## CONFLICT OF INTEREST

None declared.

## AUTHOR CONTRIBUTIONS

All authors were involved in the writing, revision, and final review of the manuscript.
